# Supplementary material for: Resolving the full spectrum of human genome variation using Linked-Reads
Source: Genome Res. 2019 Apr;29(4):635–45. doi: 10.1101/gr.234443.118 (PMC6442396; doi:10.1101/gr.234443.118)
Supplement: Supplemental Material [file supp_gr.234443.118_Supplemental_longranger-2.2.zip › longranger-2.2.tar.bz2!/lr2.2-public/lib/stan/cmdstan/stan/doc/api/html/agrad_2fwd_2functions_2lmgamma_8hpp_source.html]

Stan: src/stan/agrad/fwd/functions/lmgamma.hpp Source File


|  |  |
| --- | --- |
|  | Stan  2.5.0  probability, sampling & optimization |


- Main Page
- Related Pages
- Namespaces
- Classes
- Files

- File List
- File Members

All Classes Namespaces Files Functions Variables Typedefs Enumerations Enumerator Friends Macros Pages

- src
- stan
- agrad
- fwd
- functions

lmgamma.hpp

Go to the documentation of this file.

1 #ifndef STAN\_\_AGRAD\_\_FWD\_\_FUNCTIONS\_\_LMGAMMA\_HPP

2 #define STAN\_\_AGRAD\_\_FWD\_\_FUNCTIONS\_\_LMGAMMA\_HPP

3

4 #include <stan/agrad/fwd/fvar.hpp>

5 #include <stan/meta/traits.hpp>

6 #include <stan/math/functions/digamma.hpp>

7 #include <stan/math/functions/lmgamma.hpp>

8

9 namespace stan {

10

11  namespace agrad {

12

13  template <typename T>

14  inline

15  fvar<typename stan::return\_type<T,int>::type>

16  lmgamma(int x1, const fvar<T>& x2) {

17  using stan::math::lmgamma;

18  using stan::math::digamma;

19  using std::log;

20  T deriv = 0;

21  for(int count = 1; count < x1 + 1; count++)

22  deriv += x2.d\_ \* digamma(x2.val\_ + (1.0 - count) / 2.0);

23  return fvar<typename

24  stan::return\_type<T,int>::type>(lmgamma(x1, x2.val\_), deriv);

25  }

26  }

27 }

28 #endif

digamma.hpp

lmgamma.hpp

stan::agrad::fvar

**Definition:** fvar.hpp:13

traits.hpp

stan::return\_type::type

boost::math::tools::promote\_args< typename scalar\_type< T1 >::type, typename scalar\_type< T2 >::type, typename scalar\_type< T3 >::type, typename scalar\_type< T4 >::type, typename scalar\_type< T5 >::type, typename scalar\_type< T6 >::type >::type type

**Definition:** traits.hpp:406

stan::math::lmgamma

boost::math::tools::promote\_args< T >::type lmgamma(const int k, T x)

Return the natural logarithm of the multivariate gamma function with the speciifed dimensions and arg...

**Definition:** lmgamma.hpp:57

stan::math::digamma

double digamma(double x)

**Definition:** digamma.hpp:39

fvar.hpp

stan::agrad::digamma

fvar< T > digamma(const fvar< T > &x)

**Definition:** digamma.hpp:16

stan::agrad::fvar::d\_

T d\_

**Definition:** fvar.hpp:16

stan::agrad::log

fvar< T > log(const fvar< T > &x)

**Definition:** log.hpp:15

stan::agrad::lmgamma

fvar< typename stan::return\_type< T, int >::type > lmgamma(int x1, const fvar< T > &x2)

**Definition:** lmgamma.hpp:16

stan::agrad::fvar::val\_

T val\_

**Definition:** fvar.hpp:15


---

[ Stan Home Page ]

*© 2011–2014,
Stan Development Team.*
